# Deducing *in Vivo* Toxicity of Combustion-Derived Nanoparticles from a Cell-Free Oxidative Potency Assay and Metabolic Activation of Organic Compounds

**DOI:** 10.1289/ehp.11370

**Published:** 2008-08-22

**Authors:** Tobias Stoeger, Shinji Takenaka, Birgit Frankenberger, Baerbel Ritter, Erwin Karg, Konrad Maier, Holger Schulz, Otmar Schmid

**Affiliations:** Helmholtz Zentrum München, German Research Center for Environmental Health, Institute of Inhalation Biology, Neuherberg/Munich, Germany

**Keywords:** air pollution, BET, biotransformation, carbonaceous particles, *Cyp1a1*, dose response, nanoparticles, nanotoxicity, organic compounds, oxidative stress, particle toxicity, soot particles, specific surface area, surface toxicity, ultrafine particles

## Abstract

**Background:**

The inhalation of combustion-derived nanoparticles (CDNPs) is believed to cause an oxidative stress response, which in turn may lead to pulmonary or even systemic inflammation.

**Objective and Methods:**

In this study we assessed whether the *in vivo* inflammatory response—which is generally referred to as particle toxicity—of mice to CDNPs can be predicted *in vitro* by a cell-free ascorbate test for the surface reactivity or, more precisely, oxidative potency (*Ox*_Pot_) of particles.

**Results:**

For six types of CDNPs with widely varying particle diameter (10–50 nm), organic content (OC; 1–20%), and specific Brunauer, Emmett, and Teller (BET) surface area (43–800 m^2^/g), *Ox*_Pot_ correlated strongly with the *in vivo* inflammatory response (pulmonary polymorphonuclear neutrophil influx 24 hr after intratracheal particle instillation). However, for CDNPs with high organic content, *Ox*_Pot_ could not explain the observed inflammatory response, possibly due to shielding of the *Ox*_Pot_ of the carbon core of CDNPs by an organic coating. On the other hand, a pathway-specific gene expression screen indicated that, for particles rich in polycyclic aromatic hydrocarbon (PAHs), cytochrome P450 1A1 (CYP1A1) enzyme-mediated biotransformation of bio-available organics may generate oxidative stress and thus enhance the *in vivo* inflammatory response.

**Conclusion:**

The compensatory nature of both effects (shielding of carbon core and biotransformation of PAHs) results in a good correlation between inflammatory response and BET surface area for all CDNPs. Hence, the *in vivo* inflammatory response can either be predicted by BET surface area or by a simple quantitative model, based on *in vitro Ox*_Pot_ and *Cyp1a1* induction.

Epidemiologic and clinical studies have linked elevated concentrations of ambient particulate matter (PM) to adverse health effects throughout the industrialized world (Kleeberger 2005). In urban environments, combustion-derived nanoparticles (CDNPs) such as soot particles dominate PM number concentrations and contribute significantly to the total PM surface area. Typically, these particles consist of a carbon core coated with potentially toxic air pollutants consisting of hundreds of organic chemicals and many transition metals (Kreyling et al. 2004; Oberdörster et al. 2005). At present, it is commonly hypothesized that the toxicity of ultrafine soot particles is largely driven by adsorbed redox-active components [e.g., poly-aromatic hydrocarbons (PAHs)], which participate in redox-cycling reactions generating reactive oxygen species (ROS). These ROS can cause oxidative stress responses that may result in pulmonary or even systemic inflammation (Li et al. 2003a; Risom et al. 2005). Ultimately, these processes may promote the progression of atherosclerosis and precipitate acute cardiovascular responses ranging from increased blood pressure to myocardial infarction (Delfino et al. 2005). To counteract the adverse effects of oxidative stress generated by redox cycling and electrophilic compounds, nature “developed” an elaborate defense system to maintain redox homeostasis. This system includes a series of anti-oxidant enzymes, in particular, the so-called phase II xenobiotic metabolism enzymes that metabolize redox-cycling chemicals, eliminate excessive ROS, and protect cells against the damaging effects of electrophiles and oxidants (Winyard et al. 2005). A disturbance of the cellular redox equilibrium and related disproportionate oxidative stress is well known to trigger multiple stress kinase pathways and redox-sensitive transcription factors, such as nuclear factor-erythroid 2-p45–related factor 2 (Nrf2), which activates the expression of antioxidant and cytoprotective genes, as well as the nuclear localization of nuclear factor kappa B (NF-kB) and activator protein-1 (AP-1), which play key roles in gene promotion for inflammatory mediators (Cho et al. 2006; Rahman et al. 2006).

Identification of the particle properties that are most relevant for promoting adverse health effects is crucial not only for our mechanistic understanding but also for the implementation of strategies for improving air quality. For different kinds of particles with low toxicity and solubility, particle surface area has shown to be a useful dose metric for the characterization of particle-induced neutrophil response in the exposed lung (Oberdörster et al. 2005). Inflammatory effects of diesel exhaust particles (DEP) appear to be driven by particle surface area (Heinrich et al. 1995), although the type of organic (Baeza-Squiban et al. 1999) and metal (Ball et al. 2000) components also appear to play a role in oxidative and proinflammatory effects and subsequent pathogenicity. This is consistent with studies describing different classes of surface toxicity pointing to different pathways of particle-related stress induction (Dick et al. 2003; Donaldson et al. 2005; Duffin et al. 2002; Warheit et al. 2006).

In a previous study, we found that for six different types of CDNPs, the organic carbon content (OC) had a much weaker impact on the acute inflammatory response in the lungs of mice (24 hr after intratracheal instillation) than did Brunauer, Emmett, and Teller (BET) surface area, with predictive values of 0.0286 versus < 0.0001, respectively (Stoeger et al. 2006). Although BET surface area appears to be the single most relevant parameter for the prediction of particle toxicity, it is likely that other parameters such as the presence of free radicals, particle charge, and the bioavailability of adsorbed components may explain the observed differences in surface toxicity (Stoeger et al. 2006; Wittmaack 2007).

The objective of the present study was to assess the possibility of predicting particle toxicity based on the combined information from a cell-free *in vitro* test for the innate oxidative potency (*Ox*_Pot_) of particles and a gene expression analysis targeting inflammation, stress, and detoxification-related genes. Here we relate the results from both tests to our previously published *in vivo* data on the acute inflammatory response of mice for six types of CDNPs (Stoeger et al. 2006) and introduce a simple two-parameter model to quantitatively describe the toxicity of CDNPs from the *Ox*_Pot_ and bioavailability of organic components. Ultimately, this model may serve as an additional step toward introducing a cell-free test for nanotoxicology that is reliable enough to make animal studies obsolete.

## Materials and Methods

### Particles

In the present study we used the same six CDNP types as we described previously (Stoeger et al. 2006). Briefly, we purchased DEPs [standard reference material SRM-1650a; National Institute of Standards and Technology (NIST), Gaithersburg, MD, USA] and PrintexG (PtxG) and Printex90 (Ptx90; pigment black; Degussa, Frankfurt, Germany) and generated and filter-sampled SootH (high OC), SootL (low OC), and ultra-fine carbon particles (UfCPs) in our laboratory. We produced SootH and SootL particles by a well-controlled diffusion flame (CAST burner, Matter Engineering AG, Wohlen, Switzerland) with low and high oxygen-to-fuel ratios, respectively, resulting in soot particles with different amounts (and types) of organic compounds (Matuschek et al. 2007). The UfCPs, generated by spark discharge from graphite electrodes (GFG1000, Palas, Karlsruhe, Germany) (Roth et al. 2004), are noncombustion-derived particles with low OC, large specific surface area, and structural and compositional similarities with modern (Euro IV) diesel soot (Su et al. 2004). Table 1 lists the BET surface area [determined using the method described by Brunauer, Emmett, and Teller (1938)] and OC of all six particle types as reported previously (Stoeger et al. 2006) covering a wide range from about 40 to 800 m^2^/g and from 1% to 20% OC. The (primary) particle diameter of the nanoparticle types ranged from about 10 to 50 nm (Stoeger et al. 2006). For the biological analysis of the CDNPs described below, we used the entire particles rather than solvent extracts.

### Determination of the Ox_Pot_ of the particles

In living organisms, vitamin C (l-ascorbate), a highly effective antioxidant, is consumed as it protects the body against oxidative stress. Therefore, we used the depletion of ascorbate in an aqueous particle suspension as a cell-free *in vitro* test for the *Ox*_Pot_ of particles, where the absence of any metabolic activity in a cell-free system guarantees that this test characterizes the particles’ innate (in absence of metabolic activation) *Ox*_Pot_ presumably related to the surface reactivity of the particles.

**Figure 1:**
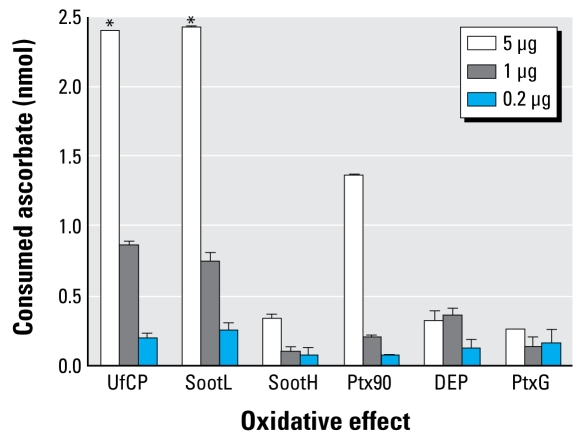
Cell-free oxidative effect (mean ± SE) of the six types of CDNP displayed as the amount of ascorbate consumed by the respective particle mass (5, 1, and 0.2 μg). *Values that were negatively biased because of their proximity to the saturation level (2.5 nmol) were discarded for the calculation of *Ox*_Pot_ (Table 1).

For each particle type, we incubated aliquots of 5, 1, and 0.2 μg for 40 min in an aqueous ascorbate solution (100 μL of 100 μM ascorbate in phosphate-buffered saline; this corresponds to 2.5 nmol ascorbate per 25 μL). After removal of the particles by centrifugation (30,000*g*/15 min/4°C), we quantified the antioxidative capacity of 25 μL of supernatant using the photochemiluminescence (PCL) method (Photochem, Analytik Jena AG, Jena, Germany), in which the fast photochemical excitation of radical formation is combined with sensitive luminometric detection (Popov and Lewin 1999). We performed two measurements for each particle type and concentration (Figure 1) and determined the particles’ innate *Ox*_Pot_, expressed as consumed ascorbate (nanomoles) per particle mass (micrograms) from the slope (not the ratio) of the measured ascorbate consumption and particle mass in order to avoid biases due to potential zero off-sets. For UfCP and SootL particles, the highest dose of 5 μg had to be discarded because a consumption of 2.4 nmol of 2.5 nmol ascorbate points to saturation issues.

### Determination of the inflammatory efficacy of the particles

In the present study, we used the *in vivo* animal exposure data from our previous study (Stoeger et al. 2006). In brief, for each particle type, we determined the response of eight female BALB/cJ mice (21.1 ± 1 g) after intratracheal instillation of 5, 20, or 50 μg CDNPs in 50 μL aqueous suspension or pure water (sham control), except for UfCP, which also included instillations with 0.5 and 2 μg particles. We treated animals humanely and with regard for alleviation of suffering; experimental protocols were reviewed and approved by the Bavarian Animal Research Authority (approval no. 211-2531-108/99). At 24 hr after instillation, we performed broncho-alveolar lavage (BAL) and assessed BAL fluid for polymorphonuclear neutrophils (PMNs) as a measure of the inflammatory cellular response. In the present study, the previously reported absolute numbers of PMNs (Stoeger et al. 2006) were normalized by the number of BAL leukocytes [Supplemental Material, Figure 1 (http://www.ehponline.org/members/2008/11370/suppl.pdf)], thus eliminating some bias due to varying effectiveness of cell retrieval by the lavage procedure (especially for 50 μg PtxG).

Using these data, in the present study we defined the (*in vivo*) inflammatory efficacy (*I*_Ef_) as the 20% PMN effect level divided by the particle mass causing this effect level (M_20%PMN_), that is, *I*_Ef_ = 20% PMN/M_20%PMN_ (%PMN/μg). We chose the 20% PMN threshold because it is significantly different from the sham response (~ 2%), it is clearly beneath saturation levels (> 40%), and it is exceeded by all six particle types [See Supplemental Material, Figure 1 (http://www.ehponline.org/members/2008/11370/suppl.pdf)]. We derived the M_20%PMN_ values by interpolation between the two PMN data points bracketing the 20% effect level.

### Quantification of gene and protein expression

For expression analysis, lavaged lungs from the 20 μg exposure were homogenized using the FastPrep FP120 Cell Disruptor (BIO101/Savant Instruments Inc., Holbrook, NY, USA) automat with Lysing Matrix Tube D (MP Biomedicals, Illkirch, France) and subjected to either RNA or protein extraction (four mice per group). RNA isolation from lung homogenates was processed using the RNeasy Kit (Qiagen, Hilden, Germany). For quantitative reverse-transcriptase polymerase chain reaction (PCR), we used 1 μg total DNase I–treated RNA for the first-strand cDNA reaction using hexamer primer (Promega, Mannheim, Germany) and Superscript II reverse transcriptase (Invitrogen, Karlsruhe, Germany). We pooled cDNA samples from each experimental group and quantified transcript levels using Absolute QPCR SYBR Green Mix plus ROX kit (ABgene, Hamburg, Germany) with the ABI Prism 7000 Sequence Detection System (Applied Biosystems, Foster City, CA, USA). We used the comparative C_T_ (threshold cycle) method to calculate the relative abundance of transcript (Applied Biosystems 1997). For normalization, we used 18S RNA [GenBank accession no. NT_039649.7 (National Center for Biotechnology Information 2008)] as the reference gene to derive the fold increase in relation to the sham control group. Details of the primer and sequence specifications are given in Supplemental Material, Table 1 (http://www.ehponline.org/members/2008/11370/suppl.pdf).

We performed extraction of cytoplasmic lung protein in RIPA buffer plus Protease Inhibitor Cocktail III (Merck Chemicals, Darmstadt, Germany). We pooled 20 μg of total lung protein from each of the eight experimental groups (six CDNP types and two controls) and separated it by sodium dodecyl sulfate gel electrophoresis using the XCell II Blot Module and NuPage Novex Bis-Tris Gels and blotted to polyvinyl difluoride membranes (Invitrogen). The following primary antibodies were used: rabbit anti-cyclooxygenase-2 (COX2; ab15191; Abcam, Cambridge, UK), rabbit anti-cytochrome P450 1A1 (CYP1A1; sc-20772; Santa Cruz Biotechnology, Heidelberg, Germany), rabbit anti-rat liver glutathione *S*-transferase α1 (GST-Ya; AB 0009; Gentaur, Aachen, Germany), rabbit anti-heme oxygenase-1 (HO1; SPA 895; Biomol, Hamburg, Germany), rabbit anti-heat shock 70-kDa protein 1B (HSP70; SPA 812, Biomol), goat anti-NAD(P)H dehydrogenase [quinone] 1 (NQO1; IMG-3033, Biomol), and, as loading control, mouse monoclonal anti–β-actin (ACTB), AC-15 (A1978; Sigma-Aldrich, Taufkirchen, Germany). We applied horseradish peroxidase–linked species-specific secondary antibody for primary antibody detection, donkey anti-rabbit IgG (Amersham/GE Healthcare, Buckinghamshire, UK), rabbit anti-goat (Chemicon International, Millipore Corp., Billerica, MA, USA), and sheep anti-mouse (Amersham/GE Healthcare), using the ECT Plus Detection System (Amersham/GE Healthcare). Scanned blots were analyzed using the Syngene software image analysis program (Syngene, Cambridge, UK) with baseline correction setting “rolling disc.” Protein expression derived from quantified densities of respective bands were normalized to ACTB and expressed as fold change relative to sham controls.

### Statistical analyses

All values are reported as the mean ± SE. We used analysis of variance calculated by Statgraphics software (STSC Inc., Rockville, MD, USA) to establish the statistical significance of differences between the experimental groups. We applied the Tukey honestly significant difference procedure to evaluate the significant differences between the exposed animals and the sham control group. We considered differences significant at *p* < 0.05.

## Results

### In vitro *Ox*_Pot_, in vivo *I*_Ef_, and surface area of the particles

**Figure 2:**
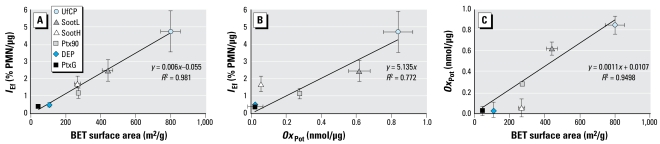
Relationships between the *in vitro Ox*_Pot_, *in vivo I*_Ef_, and BET surface area of the six types of CDNPs. (*A* ) BET surface area versus *I*_Ef_ with the linear regression line. (*B*) *Ox*_Pot_ versus *I*_Ef_ (the linear regression line was forced through the origin). (*C*) BET surface area versus oxidative potency (the linear regression was based on all data points except SootH and DEP).

As indicated in our previous study (Stoeger et al. 2006), Figure 2A confirms the excellent linear correlation between inflammatory response and BET surface area for the six types of CDNPs used in the present study. For a wide dynamic range of more than a factor of 10 (with UfCP and PtxG at the upper and lower end, respectively), the differences in BET surface area explain 98% (*R*^2^) of the observed variability in *I*_Ef_. Neglecting the small intercept of 0.055% PMN/μg, the slope of 0.006% PMN/mm^2^ indicates that, on average, a particle surface area of 33 cm^2^ is required to induce a 20% influx of PMNs into the lungs. Figure 2B shows a reduced but still strong linear correlation between the *I*_Ef_ and *Ox*_Pot_ of the particles (*R*^2^ = 0.77; *I*_Ef_ = 5.14*Ox*_Pot_; forced through the origin) with an identical order of descending *I*_Ef_ and *Ox*_Pot_ except for SootH, which has a more than three times higher *I*_Ef_ than expected from its *Ox*_Pot_. This can be interpreted as a reduced surface-specific *Ox*_Pot_, as illustrated in Figure 2C, which clearly shows SootH (and, to a lesser degree, DEP) below the trend line based on the other four data points.

### Gene expression analysis reveals bio-activation of particle-borne organics

To identify the reason for the irregular behavior of SootH, in the lungs of mice 24 hr after the instillation of 20 μg of SootH, we analyzed the expression patterns of 50 genes representing different biologic response types, including inflammation, stress response, metal detoxification, and phase I and II detoxification of xenobiotics such as PAHs.

Table 2 displays the expression of the 22 most notably regulated genes [see also Supplemental Material, Figure 2 (http://www.ehponline.org/members/2008/11370/suppl.pdf)] in terms of fold induction in relation to sham controls; that is, unity represents the absence of gene regulation. We averaged the abundances of proinflammatory marker genes (rows 2 through 9) in Table 2 for each particle type to obtain a mean score for a transcriptional proinflammatory response— “proinflammatory synopsis.” Ranking the particles in terms of the proinflammatory synopsis (Table 2) and PMN-based cellular lung inflammation (*I*_Ef_ in Table 1) shows good agreement and validates the relevance of our RNA expression data.

We gained insight into transcriptional regulations related to general stress response by examining the inducible 70-kDa heat shock gene *Hsp1a1* as well as the metallothionein 1 and 2 genes (*Mt1* and *Mt2*), all known to be up-regulated directly by oxidative stress and toxic chemicals—particularly heavy metals. Only PtxG particles showed a more than 2-fold increase in *Hsp1a1* lung transcript levels 24 hr after instillation; for the other carbon black sample (Ptx90), mRNA levels were 3-fold decreased. Gene expression of *Mt1* and *Mt2* was increased for UfCP only; for DEP, these genes were 2-fold repressed. This indicates that, although the toxicity of metals may have contributed to the oxidative stress induced by UfCP and DEP, metals do not explain the extreme deviation of SootH from the linear response curve or the general trend in Figure 2B.

The expression analysis of 11 selected detoxification enzymes (*Cyp1a1*, *Cyp1b1*, *Aldh3a1*, *Gclc*, *Gpx1*, *Gpx4*, *Gsr*, *Gsta1*, *Hmox1*, *Nqo1*, and *Ogg1*) revealed that each particle type induced at least one of these genes. However, the only gene whose expression was induced specifically by SootH (3.9-fold up-regulated) and, to a lower extent, by DEP (1.6-fold up-regulated) was the phase I xenobiotic metabolizing enzyme *Cyp1a1*. To validate these data derived from pooled samples, we also quantified *Cyp1a1* expression in SootH-instilled mice on the single-animal level (*n* = 4). Single-animal analysis confirmed our previous data detecting an induction of 4.0 ± 0.4 that is significantly (*p* = 0.001) different from sham controls [see Supplemental Material, Figure 2 (http://www.ehponline.org/members/2008/11370/suppl.pdf)]. Furthermore, low-OC particles (OC < 7%) were either not significantly affected (Ptx90, PtxG) or down-regulated (SootL, UfCP). In contrast to *Cyp1a1*, the closely related cytochrome 450 phase I enzyme isoform *Cyp1b1* did not show regulation by more than 2-fold. The investigated phase II enzymes *Gclc*, *Gpx1*, *Gpx4*, and *Gsr* were more than 2-fold induced by UfCP, but none of them was more than 2-fold induced by the OC-rich DEP and SootH particles. 8-Oxoguanine DNA glycosylase (Ogg1), a DNA repair enzyme whose expression is associated with organics-related DNA adduct formation (Sorensen et al. 2003), is more than 2-fold induced by Ptx90 and UfCP and thus also is not a suitable marker for OC-rich particle instillation.

In summary, our expression data identify *Cyp1a1* as the only marker gene whose expression is specifically induced by OC-rich particle exposure.

### OC-rich soot particles induce expression of CYP1A1 protein in the lung

**Figure 3:**
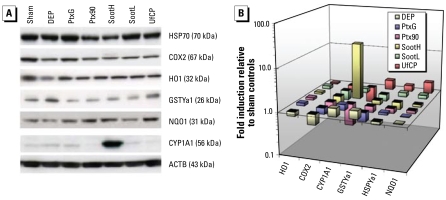
Protein expression and quantification of selected markers. (*A*) Protein expression analyzed by immunoblotting. (*B*) For quantification, signal densities were normalized to ACTB loading control and are displayed relative to the respective expressions in sham controls. CYP1A1 was the only marker that was induced for the known high-OC particles SootH (21-fold) and DEP (1.6-fold). It even was mildly induced for PtxG (1.4-fold).

Because the functional relevance of induced gene expression needs to be confirmed by a subsequent increase in expressed protein levels, we analyzed six of the investigated marker genes (*Hmox1*, *Ptgs2*, *Cyp1a1*, *Gsta1*, *Hsp1a1*, and *Nqo1*) on the protein level (HO1, COX2, CYP1A1, GSTYa1, HSP70, and NQO1) by immunoblotting of cytoplasmic extracts from lungs 24 hr after particle instillation (Figure 3). As expected from our mRNA data, only the expression of CYP1A1 protein was strongly induced by instillation of OC-rich SootH particles. Moreover, particle exposure did not influence the abundance of any of the other investigated proteins. Quantification of protein expressions relative to sham control and normalized to ACTB revealed a 21-fold induction of CYP1A1 protein expression for SootH particles and a 1.6- and 1.4-fold induction for DEP and PtxG, respectively. UfCP and Ptx90 particle instillation suppressed expression of the CYP1A1 protein to 40% and 30% of control levels, respectively. CYP1B1 was not detectable at all (not shown in Figure 3), and the phase II enzymes NQO1, GSTYa1, and HO1 did not indicate specific responses at the 24-hr time point. The inflammatory mediator COX2 (*Ptgs2*) was suppressed up to 2-fold 24 hr after instillation of all particles.

### Immunohistologic localization of CYP1A expression

**Figure 4:**
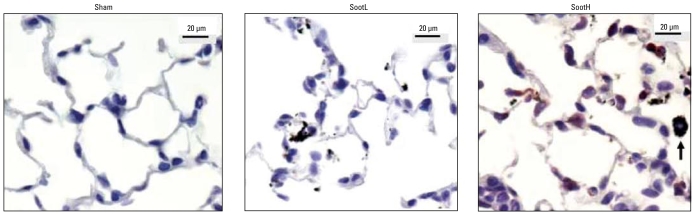
Immunohistologic staining of CYP1A1 protein expression in lungs 24 hr after 20 μg particle instillation showed broad positive staining in epithelial cells (red) and some alveolar macrophages (data not shown) of SootH-exposed mice. No staining was detected in sham- or SootL-exposed lungs. Agglomerates from SootH and SootL particles are clearly visible in the lungs. The arrow indicates a SootH-loaded macrophage containing agglomerated particles.

Immunohistologic detection of CYP1A1 protein expression in lungs 24 hr after instillation of 20 μg particles revealed the strongest expression for SootH-exposed lungs (Figure 4) and somewhat weaker signals for DEP-exposed lungs (data not shown). We identified CYP1A1-positive cells to be mainly alveolar epithelial cells, but we also identified some positive alveolar macrophages. No clear signal could be detected in lungs from untreated and sham-exposed mice or after UfCP, Printex, or SootL instillation.

## Discussion

### *Ox*_Pot_ is a strong indicator for particle-induced inflammatory effects

Several studies indicate that the *Ox*_Pot_ of the particle surface is critical for biologic responses related to inflammation (Beck-Speier et al. 2005; Li et al. 2003b; Xia et al. 2006). Because for the six types of CDNPs investigated here the *Ox*_Pot_ (based on a cell-free assay) can explain only about 80% of the observed variability in *I*_Ef_ (*R*^2^ = 0.77; Figure 2B), at least one more parameter contributes to the observed *in vivo* particle toxicity (*I*_Ef_). Furthermore, particles with high OC (DEP, SootH) display a mitigated surface-specific *Ox*_Pot_ compared with low-OC particles (Figure 2C). This could indicate that an organic coating shields the active structures at the surface of the carbon matrix and thus prevents radical formation. On the other hand, if *Ox*_Pot_ were the only pathway of inflammation, the surface-specific *I*_Ef_ of DEP and SootH should be reduced compared with low-OC particles (e.g., SootL, UfCP). Because Figure 2A does not show this (DEP and SootH do not lie significantly below the linear regression curve), the mitigating effect of the organic coating on *Ox*_Pot_ is compensated by another mechanism, which only occurs in the *in vivo* and not in the *in vitro* test.

### Biotransformation of particle-adsorbed organics contributes to inflammatory effects

The prime candidates for this additional mechanism are the enzymatic biotransformation of initially nontoxic (nonoxidizing) organic compounds into toxic derivatives and, to a lesser degree, the nonenzymatic, catalyst-mediated formation of free radicals such as hydroxyl radicals via Fenton chemistry by, for example, transition metals. The latter would not necessarily require a metabolic active cell, and the corresponding *Ox*_Pot_ would probably be detected by our ascorbate-PCL–based *Ox*_Pot_ measurement. However, the fact that metal-inducible *Mt1* and *Mt2* genes (Adams et al. 2002) were not up-regulated by DEP and SootH (Table 2) suggests no metal involvement.

On the other hand, the induced expression of both the *Cyp1a1* gene and CYP1A1 protein for SootH (3.9- and 21-fold for gene and protein, respectively) and, to a much lesser degree, for DEP (1.6-fold for both gene and protein) indicates that metabolic detoxification of organics takes place for particles with high OC. In addition, PtxG, the particle type with the lowest OC (1%), also shows a small induction (1.2- and 1.4-fold for gene and protein, respectively). All other low-OC particles show a down-regulation in both the *Cyp1a1* gene and CYP1A1 protein. Hence, it is obviously not only the amount of organics, but also the type of organics, that determines the regulation of *Cyp1a1*.

Pulmonary expression of *Cyp1a1* has previously been identified as a possible bio-marker of exposure to DEPs *in vitro* and *in vivo* (Bonvallot et al. 2001; Takano et al. 2002). For instance, Takano et al. (2002) instilled DEP from a light-duty automobile source and observed *Cyp1a1* transcript levels to be about 5-fold induced 24 hr after instillation of 50 μg DEP per mouse, whereas no effect was detected for 10 μg. (Bonvallot et al. 2001, Takano et al. 2002). This is consistent with results from our present study, in which instillation of 20 μg DEP from a heavy-duty diesel engine (SRM 1650a) mildly induced the *Cyp1a1* expression on RNA and protein level (1.6-fold).

Two main families of organic compounds that are known to be adsorbed onto ambient CDNPs are PAHs and quinones (Health Effects Institute 2002). After cellular uptake, PAHs could be desorbed from the CDNPs, become bioavailable to bind to the cytosolic aryl hydrocarbon receptor (AHR) and thereby induce target gene expression such as that of phase I metabolizing cytochrome P450 enzymes. The toxicity of many PAHs is known to depend on their bioactivation by P450 enzymes (Ramadoss et al. 2005). The hypothesis of PAH-related bioactivation of toxic compounds for SootH and DEP (as well as for PtxG) is supported by the chemical analysis of the particles. Both liquid chromatography and thermoanalytical investigation have revealed significant amounts of PAHs for SootH (~ 1%), exceeding those from SootL by a factor of about 50 (Schroeppel et al. 2003). Moreover, detailed chemical speciation reveals the presence of aromatic compounds such as benz(*a*)anthracene, benzo(*k*) fluoranthene, and/or benzo(*a*)pyrene, in significant amounts only on SootH, DEP, and PtxG samples (Matuschek et al. 2007; NIST 2000), which have been described as potent inducers of *Cyp1a1* (Till et al. 1999).

### Mechanistic considerations

PAH metabolism by *Cyp1a1* produces reactive electrophilic metabolites, including ROS (Baulig et al. 2003; Martignoni et al. 2006). In the present study, we did not directly quantify ROS formation in the lungs of instilled mice, but we quantified several proinflammatory genes that are known to be regulated by redox-sensitive transcription factors such as NF-kB and AP-1 (Cho et al. 2006; Rahman et al. 2006). *In vivo* we found the lung transcripts for the cytokines *Csf2*, *Cxcl1*, *Cxcl5*, and *Il6*—all known to be largely regulated by NF-kB and AP-1 (reviewed by Castranova 2004)—to be induced by SootH and SootL instillation at a similar level (Table 2), whereas the *in vitro Ox*_Pot_ of SootL exceeded that of SootH by a factor of about 6 (Figure 2B). Again, this suggests that two different mechanisms are responsible for the toxicity of SootL and SootH particles, one related to the particles’ innate *Ox*_Pot_ and the other possibly to bioactivation of organics.

Interestingly, compared with sham controls, mice instilled with SootL and UfCP had *Cyp1a1* expression that was about 3-fold down-regulated; these are the two particle types with the highest innate *Ox*_Pot_, and the very same particles caused the strongest expression of the proinflammatory mediators (*Cxcl1*, *Cxcl5*, and *Il6*), which are regulated by redox-sensitive transcription factors. Hence, the transcriptional repression of *Cyp1a1* might be explained by inhibition of *Cyp1a1* RNA expression by oxidative stress in general. This interpretation is consistent with the autoregulatory mechanism described by Morel et al. (1999) in a human hepatoma cell line; they found that intracellular ROS production disturbs the AHR-dependent induction of *Cyp1a1* expression, which represents a protective negative feedback loop. Alternatively, Gharavi and El-Kadi (2005) showed in murine hepatoma cell lines that *Cyp1a1* is down-regulated by the proinflammatory cytokines interleukin 1β (Il-1β), IL-6, and tumor necrosis factor α in an AHR-dependent manner. Even though both models address the liver and not the lung as studied here, this autoregulatory, ROS-protective mechanism could explain our observation that CDNPs with high innate *Ox*_Pot_ induce significant down-regulation of *Cyp1a1* expression.

*Cyp1a1* and *Cyp1b1*, two closely related and essential extrahepatic phase I P450 monooxygenases, are highly inducible in the human lung by certain aromatic compounds, such as benzo(*a*)pyrene, via AHR signaling (reviewed by Martignoni et al. 2006). However, with our instillation approach we observed no dependence of *Cyp1b1* mRNA expression on organic compounds, because expression levels relative to controls were weakly induced by SootH to an expression level that is identical to that of SootL (1.4-fold). This apparent contradiction to the high expression of *Cyp1a1* by SootH can be resolved if one considers that despite their close functional relationship, the CYP1A1 and CYP1B1 isoforms are regulated in a different manner. For example, *Cyp1a1* transcript expression is repressed by proinflammatory cytokines (Gharavi and El-Kadi 2005), whereas *Cyp1b1* expression is induced (Piscaglia et al. 1999). In this context, cAMP response elements as well as AP-1 sites—both frequently involved in proinflammatory signaling—have been identified in the murine *Cyp1b1* but not *Cyp1a1* promoter (Zheng and Jefcoate 2005).

**Figure 5:**
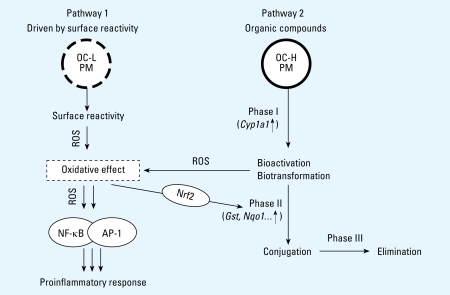
Mechanistic model for particle-related proinflammatory response that is consistent with our *in vivo* and *in vitro* data for CDNPs. The inflammatory signaling cascade is activated by oxidative stress due to the combined effects of the particles’ innate surface reactivity (measured as *Ox*_Pot_; pathway 1) and the presence of bioavailable organic compounds (*Cyp1a1* induction; pathway 2), which are eliminated via a three-phase detoxification process. Abbreviations: H, high; L, low; PM, particulate matter.

In summary, our results support the following mechanistic scheme (Figure 5) for particle-related inflammation: On the one hand, particles with high innate *Ox*_Pot_ (linked to low OC) generate direct oxidative stress by particle–cell interaction, which in turn activates redox-sensitive transcription of proinflammatory genes (pathway 1). On the other hand, particles with high amounts of bioavailable OC, such as PAHs, first induce phase I detoxification enzymes—in particular, CYP1A1 in exposed cells (pathway 2). Increased activity of CYP1A1 monooxygenase in turn leads to biotransformation and bioactivation of PAHs with associated ROS formation. Consequently, the induced oxidative stress activates transcription of both proinflammatory and phase II detoxification genes. The latter facilitate metabolization and conjugation of the highly active transformed organic compounds, ultimately leading to their elimination from the organism (Köhle and Bock 2007).

### Quantitative model for *I*_Ef_

The analysis presented here can be used to derive a simple, quantitative model for predicting the *in vivo* toxicity of CDNPs based on a simple *in vitro* assay for *Ox*_Pot_ and (*in vivo*) *Cyp1a1* gene or protein expression. As a first approach, we performed a linear regression (forced through the origin) with the ascorbate-based *in vitro* test (*Ox*_Pot_) as independent and *I*_Ef_ as dependent parameters to obtain


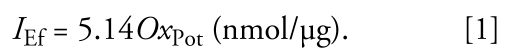


**Figure 6:**
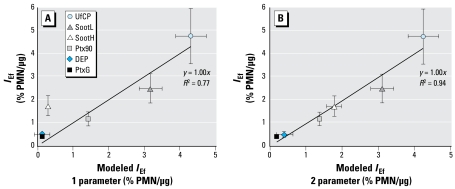
The predictive capacity of two simple linear models for the measured *I*_Ef_. Although the one-parameter regression model based on *Ox*_Pot_ only (*I*_Ef_ = 5.14*Ox*_Pot_) represents 77% of the observed variability in *I*_Ef_, the two-parameter model (*Ox*_Pot_ and *Cyp1a1* induction; see Equation 2), which considers the combined effects of pathway 1 and pathway 2, explains about 94%.

As a second approach, we modeled the *in vivo I*_Ef_ as linear function of both *Ox*_Pot_ and *Cyp1a1* gene expression (GE_Cyp1a1_ expressed as fold induction after instillation of 20 μg particles), yielding


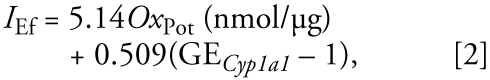


where we set *GE_Cyp1a1_* to unity (no contribution from pathway 2), if *Cyp1a1* was down-regulated (< 1-fold). As shown in Figure 6A, the one-parameter model (pathway 1 only; Equation explains only 77% of the observed variability in *I*_Ef_, whereas the two-parameter model, which considers contributions from pathways 1 and 2 (Equation 2), explains 94%. This indicates that both pathways contribute to the observed *I*_Ef_. Because the small data set (six data points) required limiting the number of independent fit parameters, we reduced the number of fit parameters to two by assuming linearity (through the origin) and independence of pathways 1 and 2 (see Equation 2). Although this is likely to be only a crude approximation for complex biological systems, the agreement between measured and modeled *I*_Ef_ is remarkable.

Because we derived our expression data from *in vivo* experiments, this model for toxicity prediction still requires animal exposures, although at a reduced amount. However, if future research will provide a means of obtaining *Cyp1a1* expression data from the exposure of cell lines, the nanotoxicologic model we present here may provide a true alternative to animal exposures.

## Figures and Tables

**Table 1:** Physicochemical and biological particle properties.

Particle	BET surface area (m^2^/g)	Percent OC (NIOSH 5040)^a^	*I*_Ef_ (% PMN/μg)	*Ox*_Pot_ (nmol/μg)^b^	*R*^2^^c^
UfCP	800	17 (< 5)^d^	4.8	0.839	1.00^e^
SootL	441	7	2.5	0.617	1.00^e^
SootH	268	19	1.7	0.057	0.99
Ptx90	272	2	1.2	0.278	1.00
DEP	108	20^f^	0.5	0.026	0.27^g^
PtxG	43	1	0.4	0.024	0.88

a The organic carbon fraction was determined with a thermooptical method according to the National Institute for Occupational Safety and Health (NIOSH) 5040 standard protocol (Cassinelli and O’Connor 1998).

b *Ox*_Pot_ from the slope of the consumed ascorbate versus particle mass (see Figure 1).

c R^2^ gives the significance (goodness of fit) of the linear regression.

d The NIOSH 5040 value of 17% (Stoeger et al. 2006) is unrealistically high for spark-generated (pure) carbon particles; more detailed chemical analysis suggests that UfCPs contained < 5% of organic matter (Frampton et al. 2004; Matuschek et al. 2007).

e Here *R*^2^ = 1 because only two data points were available for UfCP and SootL because of saturation issues (see Figure 1).

f This value was not obtained according to the NIOSH protocol; it refers to the solvent (Soxhlet)-extractable mass fraction as reported by NIST (2000).

g The low *R*^2^ value is at least in part due to the small effect level for DEP.

**Table 2:** Gene expression analysis (fold change relative to sham control) showing the mean transcript abundance assessed by quantitative PCR in the lungs of mice 24 hr after instillation of six types of CDNPs (20 μg).

Pathway	Gene	DEP	PtxG	Ptx90	SootH	SootL	UfCP
Proinflammatory	*Csf2*	2.0	2.0	5.2	5.7	6.5	3.4
	*Cxcl1*	0.7	1.9	4.0	5.6	7.5	15.9
	*Cxcl5*	0.4	2.5	2.4	4.9	4.4	9.6
	*Il6*	1.0	1.5	1.7	3.0	5.5	5.6
	*Il1b*	0.9	1.1	1.7	1.8	1.8	1.0
	*Mmp9*	0.8	1.6	1.9	2.4	1.7	2.6
	*Ptgs2*	1.1	2.1	1.4	1.4	1.3	1.1
	*Tnfa*	1.3	0.9	1.6	1.8	2.1	0.9
Proinflammatory synopsis		1.0	1.7	2.5	3.3	3.9	5.0
Stress response	*Hspa1a*	0.6	2.6	0.3	0.7	1.0	1.5
	*Mt1*	0.5	1.6	0.9	1.4	1.1	4.4
	*Mt2*	0.4	0.8	1.1	1.1	1.3	3.6
Detoxification, phase I	*Cyp1a1*	1.6	1.2	0.9	3.9	0.4	0.4
	*Cyp1b1*	0.6	0.7	0.8	1.4	1.3	0.7
Detoxification, phase II	*Aldh3a1*	0.4	0.5	0.6	1.0	0.6	0.5
	*Gclc*	0.8	2.4	1.5	1.5	1.9	4.3
	*Gpx1*	0.9	1.4	1.2	1.3	1.3	3.1
	*Gpx4*	0.6	0.8	0.9	0.8	0.8	3.1
	*Gsr*	0.7	2.1	1.3	1.2	1.3	2.5
	*Gsta1*	0.5	0.5	0.6	1.0	0.7	0.5
	*Hmox1*	1.1	1.8	1.6	1.9	1.9	0.9
	*Nqo1*	1.1	2.2	2.0	1.9	1.7	0.9
	*Ogg1*	1.3	1.9	2.8	1.9	1.5	2.5

We examined 50 genes related to various cellular response pathways, with the 22 most notably changed included here. Averaging expression levels for the eight genes indicative for the inflammatory pathway (proinflammatory synopsis) provides a proxy for the severity of inflammation.
